# The Role of Lipid Environment in Ganglioside GM1-Induced Amyloid β Aggregation

**DOI:** 10.3390/membranes10090226

**Published:** 2020-09-09

**Authors:** Vladimir Rudajev, Jiri Novotny

**Affiliations:** Department of Physiology, Faculty of Science, Charles University, 128 00 Prague, Czech Republic; rudajev@natur.cuni.cz

**Keywords:** gangliosides, GM1, amyloid β, amyloid oligomers, fibrils, Alzheimer’s disease, membrane microdomains

## Abstract

Ganglioside GM1 is the most common brain ganglioside enriched in plasma membrane regions known as lipid rafts or membrane microdomains. GM1 participates in many modulatory and communication functions associated with the development, differentiation, and protection of neuronal tissue. It has, however, been demonstrated that GM1 plays a negative role in the pathophysiology of Alzheimer’s disease (AD). The two features of AD are the formation of intracellular neurofibrillary bodies and the accumulation of extracellular amyloid β (Aβ). Aβ is a peptide characterized by intrinsic conformational flexibility. Depending on its partners, Aβ can adopt different spatial arrangements. GM1 has been shown to induce specific changes in the spatial organization of Aβ, which lead to enhanced peptide accumulation and deleterious effect especially on neuronal membranes containing clusters of this ganglioside. Changes in GM1 levels and distribution during the development of AD may contribute to the aggravation of the disease.

## 1. Introduction

Alzheimer′s disease (AD) is the most common neurodegenerative disorder responsible for 70% of all dementia cases [1]. The number of individuals with AD increased from 21.7 million worldwide in 1990 to 46.0 million in 2015 [2]. The World Alzheimer Report 2019 estimates that there were over 50 million people living with dementia in 2019, and predicts that the number will increase to 152 million by 2050 (Alzheimer’s Disease International, 2019). Clinical manifestations of AD include memory loss, cognitive decline, behavioral, and neuropsychiatric symptoms [1,3]. AD develops slowly from a preclinical phase to mild cognitive impairment. The disorder finally progresses into a fully expressed clinical syndrome characterized by the presence of intracellular neurofibrillary tangles and extracellular amyloid plaques in the human brain [4,5].

Amyloid plaques origin from the accumulation and deposition of amyloid β (Aβ). The amyloid hypothesis postulates that accumulation of Aβ in the brain is the primary event driving AD pathogenesis [6]. Oligomerization, fibrillization, and deposition of Aβ peptides may cause synaptic dysfunction, brain inflammation, and oxidative stress, and disrupt neuronal ion homeostasis and alter the balance of protein kinase/phosphatase activities, thereby leading to selective neuronal loss [7]. During the development of AD, amyloid plaques are found only in specific regions of the brain, especially in the cerebral cortex and hippocampus. Aβ fibrils start to accumulate already in the preclinical stage of AD and begin to affect brain functions [8]. Interestingly, Aβ is not toxic to the majority of cells and tissues in the body. This points to the significance of the local environment, in particular the composition and phase organization of the plasma membrane, in promoting Aβ toxicity [9,10].

Numerous studies have shown that Aβ binding to neuronal cell membranes cause deleterious effects [11,12,13]. It was suggested that Aβ induces disturbances in calcium homeostasis by forming transmembrane channels [14,15,16,17,18]. Not only direct effects of Aβ on lipid bilayer, but also changes in activities of membrane-bound receptors and channels have been observed in brains of AD patients and model animals. Glutamatergic neurons located in the hippocampus and in the frontal, temporal and parietal cortex are the most impacted by Aβ, which is reflected by altered permeability of N-methyl-D-aspartic acid (NMDA) and metabotropic glutamate receptors [12,19,20]. Similarly, cholinergic neurons of basal forebrain are also damaged by oligomeric forms of Aβ [3,21]. The cholinergic system including acetylcholine production, synaptic release and degradation, as well as acetylcholine interaction with nicotinic and G protein-coupled receptors, is a crucial player in the development of AD [22,23]. It was observed that Aβ overproduction in transgenic mice and Aβ treatment of CHO cells attenuated muscarinic acetylcholine receptor-mediated transmission [24,25,26]. Inhibitors of cholinesterase, which is responsible for acetylcholine degradation, can increase acetylcholine levels in the synaptic cleft and partially ameliorate cognitive impairment in patients with mild to severe AD [27].

Many interaction partners of Aβ have been identified among membrane proteins. Aβ binds to p75 neurotrophin receptor, the low-density lipoprotein receptor-related protein, cellular prion protein (PrPc), metabotropic glutamate receptors, nicotinic acetylcholine receptor, NMDA receptor, β-adrenergic receptor, erythropoietin-producing hepatoma cell line receptor, and paired immunoglobulin-like receptor B (reviewed in [28,29]. Not only proteins, but also membrane lipids represent a noticeable platform for Aβ binding. Aβ interactions with the plasma membrane are localized to lipid rafts and microdomains [30,31,32,33]. Specific Aβ-lipid recognition plays a role, where cholesterol, sphingomyelin, and ganglioside GM1 are supposed to be the most important factors regulating Aβ–membrane binding [10,34,35]. Such interactions may have impact on Aβ secondary, tertiary, and quaternary structure that play a role in enhancing Aβ peptide cytotoxicity [10,16,36].

## 2. Amyloid β

### 2.1. Aggregates

Amyloid plaques are composed of Aβ peptide derived from the integral membrane amyloid precursor protein (APP) [37]. The predominantly occurring forms are Aβ40 and Aβ42, which contain 40 or 42 amino acids, respectively. Aβ42 is known to be the more fibrillogenic and toxic form of Aβ. The Aβ42/Aβ40 ratio in a healthy brain is 1:10. An increased Aβ42/Aβ40 ratio is associated with familial forms of AD [32,38,39,40].

**Figure 1:**
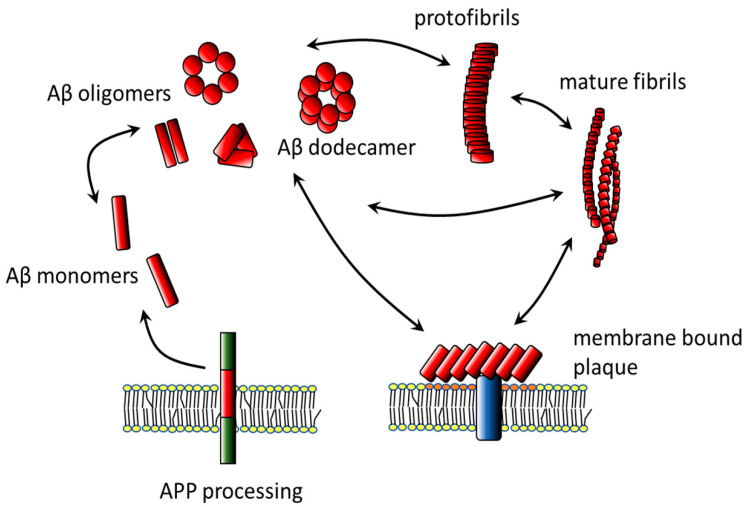
Amyloid β oligomerization. After processing of amyloid precursor protein (APP) by secretase enzymes, Aβ monomers are released into the intercellular space. In dependence on the environment, Aβ form and concentration, monomers may aggregate into supramolecular structures including low and high-molecular clusters. Among them, the 56 kDa Aβ dodecamers show the highest extent of neurotoxicity. Amyloid oligomers may form either globular, or fibrillar conglomerations known as protofibrils and fibrils. Membrane bound fibrils organize into amyloid plaques. Aβ clustering and fragmentation are reversible processes, so mutual interconversions between particular forms occur.

After being formed from APP, Aβ monomers are secreted from the cells into the interstitial space. In dependence on the concentration and the environment, Aβ42 has a propensity to assemble and form soluble aggregates, as well as ordered amyloid fibers. Soluble Aβ aggregates are generally referred to as protofibrils or oligomers. Protofibrils are elongated and show a curvilinear appearance, while globular aggregates exhibit spherical or annular morphology [41,42,43,44,45]. There is an equilibrium between monomers, oligomers, and long Aβ fibrils (Figure 1). The assembly state of the peptide plays an important role in its toxic capacity [46,47,48].

Whereas the presence of fibrillar amyloid plaques is not connected to the severity of AD pathology, the fibrils might damage the cells either directly by interacting with membranes or indirectly by acting as a source of cytotoxic amyloid forms [49]. Amyloid peptides aggregate into distinct oligomer species with different toxicities and relationships to fibrils that can be reversibly interconverted. Aβ toxicity is mostly related to the capacity of intermediate oligomers in the 5–20 nm range of dimensions to disrupt membrane integrity of neural cells [44,47,50,51,52,53]. In particular, dodecamers of Aβ42 (molecular mass of 56 kDa) represent the most toxic form. Distinct oligomeric Aβ species exert different effects on neural processes [12,51,54,55,56]. The low molecular weight forms of Aβ are considered to be less toxic [57]. Interestingly, the nondemented subjects with Aβ plaque pathology were found to have much lower oligomer-to-plaque ratios in aqueous cortical lysates than the mildly demented AD patients [58]. The plaques can sequester soluble oligomers until they reach a limit, after which excess oligomers diffuse and bind to surrounding neuronal membranes [59].

Amyloid plaques, fibrils, protofibrils, and oligomers of various size display a relatively high polymorphic variations, which is closely related to their biological effects. The monomers and oligomers of Aβ self-associate into larger structures that inherit the morphologies of nucleation centers [60,61]. Using solid state nuclear magnetic resonance (NMR) measurements, Quiang et al. revealed structural heterogeneity and qualitative difference between Aβ40 and Aβ42 aggregates in AD brain tissue [62]. However, though relatively rare, the cross-seeding of Aβ40 and Aβ42 fibrils has been observed as well [43]. Both morphology and molecular structure of Aβ are self-propagating and lead to different Aβ fibril organizations and toxicities. The morphology of Aβ supramolecular assemblies is sensitive to subtle differences in fibril growth conditions, such as pH, peptide concentration, and lipid environment [63,64]. When analyzing amyloid plaques, Rasmussen et al. [65] observed that Aβ can aggregate as clouds of conformational variants that differ among certain subtypes of AD. Aβ42 exhibits a higher aggregation propensity, and induces greater toxicity in cultured neurons as compared with the more prevalent but slower aggregating Aβ40 [4]. The hydrophobic C-terminal amino acids of Aβ42 play a crucial role in Aβ oligomer or fibril formation [4,46].

### 2.2. Secondary and Tertiary Structure

Aβ has been characterized as an intrinsically disordered peptide containing a mixture of secondary structures, both in experiments and simulations [33,66]. Results with Aβ40 identified the monomer to oligomer transition as a fundamental step of the conformational change of the peptide that is associated with the increased membrane affinity and neural toxicity [67]. Aβ peptides have a propensity to organize into helices or β-sheet containing hairpins or extended forms, where solvents play a role in fine tuning of the structure. Wei and Shea [68] showed that the monomeric state of Aβ25-35 adopts a β-hairpin conformation in water and a helical conformation in lipid mimicking solvents.

If, in the monomeric form, Aβ peptide folds into soluble random coil with some transient β-sheet or α-helical structure, whereas, in the aggregated form, a less soluble β-sheet-rich structures were observed in brain [69]. Using NMR spectroscopy, Shao et al. [70] established that the α-helix is the predominant structural feature in SDS solutions. On the other hand, simulations of Aβ25-35 showed that the monomer preferentially forms a β-hairpin [66]. However, a transition from compact β-hairpin conformations to extended β-strand structures may occur between dimeric and trimeric forms of amyloid peptide. Another NMR spectroscopy study, surface plasmon enhanced Raman spectroscopy, and molecular dynamics (MD) simulations showed a significant α-helical content in the Aβ40 monomer. During oligomerization, the secondary structure changes into a sticky conformation rich in β sheets [70,71,72].

Using a combination of soft-touch atomic force microscopy (AFM), size exclusion chromatography and native gels, Ahmed et al. have shown that, depending on the environment, different oligomers are formed. In water solution, stable disc-shaped pentamers associate with fibrils, whereas Aβ42 dodecamers are found in lipid environments [39]. Molecular dynamics simulations demonstrated Aβ monomers binding to the dimyristoyl-phosphatodylcholine (DM-PC) bilayer that leads to structural transition by forming stable helix structure in its C-terminal, which penetrates into the bilayer hydrophobic core [73].

### 2.3. Variability

**Figure 2:**
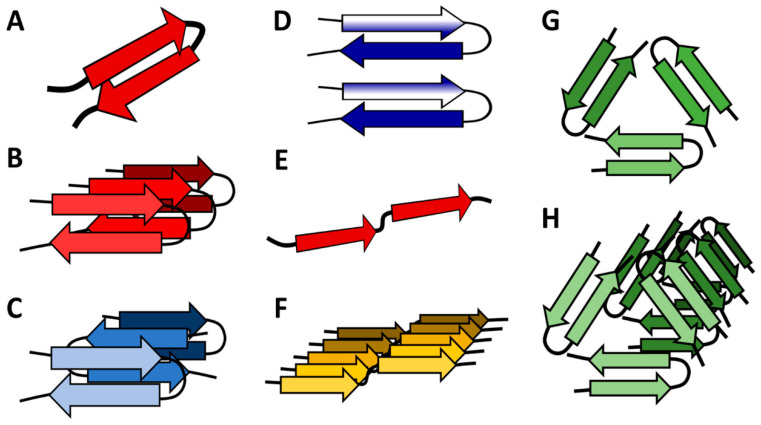
β-Sheet-containing forms of amyloid β. In dependence on the environment and peptide concentration, Aβ may organize into distinct combinations of β-rich tertiary and quaternary structures. (**A**) β-hairpin; (**B**) parallel β-hairpin structure. Particular peptides are interconnected through non-covalent interactions; (**C**) antiparallel arrangement, characteristic for toxic oligomers of amyloid peptide; (**D**) different mutual positions of internal β-sheets result from rotation of the upper part of the amyloid peptide; (**E**) an extended conformation of β-sheets containing amyloid monomer; (**F**) structure of amyloid fibril with parallel orientation of Aβ monomers. (**G**) Supposed organization of a trimer; (**H**) organization of a fiber formed of trimers. Many other possibilities of fibrillar and globular aggregates including pentamers and hexamers were described, but are not shown here. Adjusted according to [39,41,61,74].

Different interactions between Aβ monomers are reflected by changes in the secondary and tertiary structure of the peptide. Each Aβ peptide may form a β-sheet and two Aβ peptides can organize into a β-sheet dimer. The dimer interactions include several variables reflecting the internal arrangement of β-strand monomer and spatial interactions between particular peptides [32,74] (Figure 2). Molecular dynamics simulations and comparisons with AFM images led to characterization of transmembrane β-barrels forming membrane channels. These contained parallel β-strands, where the strands of each monomer were connected turn by turn [75].

Detailed comparison of the Aβ42 and Aβ40 fibril structures revealed that they share a similar protofilament structure [76]. However, despite the minimal sequence difference, Aβ42 folds into fibril having a distinct tertiary fold from those observed for Aβ40 fibrils. The atomic model of Aβ42 amyloid fibril based on solid-state NMR data displays parallel β-sheet segments that are different from structures of Aβ40 fibrils. Ala42 in the carboxyl terminus, absent in Aβ40, forms a salt-bridge with Lys28 as a self-recognition molecular switch that excludes Aβ40 from amyloid propagation machinery [77]. Barz et al. observed that Aβ42 forms more contacts between the hydrophobic C-termini than Aβ40 [78]. Aβ42 preferentially forms parallel, in-register β-sheets that perpetuate along the fibril axis [38,63]. The morphology of the oligomers organized into anti-parallel β-sheets seems to be a fingerprint of the toxic species, whereas parallel β-sheets occur in the form of long fibers [38,39,48,79,80]. The anti-parallel organization can favor fibril fragmentation, which may result in the formation of smaller aggregates that are more deleterious to neural cells [49,79] and may be responsible for membrane permeation [48].

It has been observed that the lateral association of Aβ42 is correlated with the conversion of random coil structure into β-sheets. The intermediate step includes the antiparallel β-hairpin formation in Aβ42 oligomers. The hydrophobic effect drives the initial association of the hydrophobic sequences in the Aβ peptides, and then the β-sheet is stabilized through intermolecular hydrogen bonds. The conversion to fibrils involves the untangling of the hydrophobic regions to first form an antiparallel β-hairpin structure. The strand rotation follows leading to the parallel β-sheet structure, as the hairpins are not seen in the fibrils. During increasing Aβ concentrations, a transient antiparallel β-hairpin structure is associated with neuronal toxicity [41,66]. Interestingly, the rate of structural transformation of Aβ40 is higher compared to that of Aβ42, so Aβ40 seems to be more flexible than Aβ42 [13]. Aβ42 exhibits a greater β-strand propensity than Aβ40 [41].

### 2.4. Interactions of Aβ with the Membrane

#### 2.4.1. Membrane Binding

Even in brains of AD patients, the cerebrospinal fluid Aβ concentration (3–8 nM in healthy individuals and 3 times more in AD brains) is several orders of magnitude below the micromolar limit required for amyloid peptide aggregation. Thus, there must be a mechanism which would facilitate the aggregation process, and cellular membranes appear to play a crucial part in this mechanism [16,32,81,82]. The membrane binding is essential for Aβ to express cytotoxicity that is specific for certain brain regions and resides in distinctive cell characteristics [83].

There are two types of Aβ–membrane interactions. The Aβ peptide may insert into the membrane and form a pore or it stays attached to the surface of the membrane. The binding of Aβ may compress the membrane and make it thinner [41]. The negatively charged membrane surface may play a role in these interactions. When the lipid bilayer contains negative charge bearing lipids, Aβ can bind electrostatically via its positively charged amino acid residues. However, if the surface potential increases, the hydrophobic effect drives the peptide to insert into the membrane [64]. Once soluble and unstructured Aβ binds to the membrane, the peptide changes its conformation and forms α-helical transmembrane pores or β-structured fibrils [84,85]. Not only the presence of negatively charged lipids, but also the curvature, phase organization, and rigidity of the membrane are critical parameters determining the mode of Aβ–membrane interaction. The more fluid the membrane is, the easier is incorporation of Aβ between lipid molecules [64,86]. A substrate-supported planar bilayer model by Sasahara et al. [87] have demonstrated that the fluidity of the bilayer significantly decreases after the binding of Aβ. Hence, the relationship is bidirectional. Whereas relatively rigid membranes induce the β-sheet-rich conformation state of Aβ and its aggregation on the membrane surface, the amyloid peptide enhances membrane rigidity. Moreover, the membrane-anchored aggregates of Aβ are apparently different from those formed in solution [87]. The same membrane which is responsible for Aβ binding and aggregating is directly damaged by thinning, forming transmembrane pores or other cytotoxic arrangements of the Aβ peptide [86].

#### 2.4.2. Aβ–Lipid Interactions

Even in brains Aβ binding to the membrane requires specific intermolecular interactions, as Aβ assembles only in certain regions of the brain. Aβ peptide neurotoxicity may be mediated, at least in part, by direct interactions between Aβ and membrane lipids. Amyloid peptides are known to bind specifically to membranes enriched in cholesterol [17,88,89,90] and sphingolipids, mainly sphingomyelin and ganglioside GM1 [35,91,92]. It was demonstrated that cholesterol concentration influences the morphology and aggregation state of Aβ peptide. In the presence of cholesterol, Aβ prefers to stay at the membrane surface mainly in a β-sheet-rich conformation, but when the ratio of cholesterol to phospholipids rises Aβ can insert spontaneously into the lipid bilayer in the form of α-helix-rich oligomers. The consequence of Aβ–cholesterol interaction is a conformational change that forces Aβ to adopt a tilted orientation favorizing the aggregation into annular pores rich in α-helical structures [14,17,36,88]. On the other hand, computational modeling and MD studies revealed that cholesterol induces higher β-sheet content in the Aβ peptide oligomers, which may lead to faster fibril formation [93]. After contacting the membrane, intrinsically disordered monomers of Aβ undergo a series of lipid induced conformational changes, leading to the formation of oligomers. The aggregates may be rich in β-sheet structures (membrane pores, amyloid fibrils) or in α-helical structures (transmembrane channels) [16].

Using liposomes as model membranes, Wong et al. [9] demonstrated that bilayer phase and fluidity did not affect the Aβ binding. However, acyl chain saturation and cholesterol content were critical for the permeabilization activity of the peptide. It was also shown that soluble amyloid peptide oligomers, but not fibrils, were the primary membrane binding species. The interaction with negatively charged membranes increased Aβ oligomerization and induced the transition of amyloid peptide from random to either β-sheet or α-helical structure. Additionally, as the ratio of lipid/peptide increased, the β-sheet content diminished and the peptide was converted to mostly α-helical structure. On the other hand, only β-sheet, and not α-helix-rich structures, were connected to increased Aβ oligomerization [9]. Davis et al. described the effect of local pH related to the presence of anionic lipids on Aβ oligomerization and accumulation on the membrane surface [81]. McLaurin et al. [84] demonstrated that Aβ40/42-induced disruption of acidic lipid membranes was more pronounced at pH 6 than at pH 7. The role of pH in aggregating process seems to be of great importance because Aβ causes neurotoxicity by disrupting endosomal membranes leading to endo/lysosomal dysfunction [94].

On the basis of MD simulations, Yu and Zheng [95] suggested that charged lipid headgroups act as anchors for the initial binding of Aβ by electrostatic interactions. Then, hydrophobic residues of the peptide are locked on the bilayer by additional hydrophobic interactions. Inclusion of cholesterol makes this binding process more energetically favorable.

#### 2.4.3. Aβ and Membrane Microdomains

As mentioned above, Aβ oligomers bind to the plasma membrane predominantly at the sites where ganglioside GM1, sphingomyelin (SM), and cholesterol are concentrated [96,97]. Such lipid composition is typical for membrane structures known as membrane microdomains or lipid rafts. Membrane microdomains differ significantly from non-raft membranes as to the presence of relatively high cholesterol and saturated long chain lipid molecules content (especially sphingolipids). Raft lipids usually prefer liquid ordered phase, which is more organized and less fluid than the rest of the cell membrane [98,99,100,101,102]. In any case, membrane microdomains are dynamic structures that can coalesce to form larger platforms and separate into smaller ones. There is evidence of considerable heterogeneity in the protein and lipid composition of lipid rafts, including variation in ganglioside and sterol composition that is dependent on the tissue and cell type [103,104]. A vast number of membrane proteins participating in signaling processes have been found to associate with lipid rafts: some tyrosine kinases of the Src family, G protein-coupled receptors, trimeric G proteins and their effectors, ion channels, surface GPI-anchored molecules, etc. [105,106,107,108,109,110]. In addition, Aβ binding proteins, e.g., PrPc and glutamate receptors, are concentrated in membrane microdomains [111,112].

It was demonstrated that rigid membranes containing SM may facilitate the conversion of Aβ peptides to a β-sheet-rich form after binding to the membrane surface. The rigidity of SM-rich membrane reduced the interactions of Aβ42 with the bilayer, thereby mediating its transition to a β-sheet-containing structure not observed in the other bilayers [33]. Raft-residing gangliosides (GM1) were found to play a crucial role in Aβ binding and toxicity [91]. On the other hand, Aβ binds also to non-raft membrane regions, as was observed in artificial membrane structures [112,113]. If only liquid-ordered (Lo) and liquid-disordered (Ld) phases are present in ternary mixtures of dioleoyl-phosphatidylcholine, SM and cholesterol, the Aβ peptide prefers to bind to Ld phase, as was demonstrated using AFM [113]. However, such an interaction is relatively weak. Inclusion of GM1 led to enhanced Aβ aggregation. Interestingly, the types of aggregates differed in dependence on lipid composition. GM1 apparently catalyzed amyloid peptide aggregation, which seemed to induce both membrane disruption and fibrillogenesis [114]. Besides the fluidity and specific lipid–Aβ interactions, the curvature of membranes seems to play a significant role. The more curved or disturbed the membrane is, the stronger the amyloid aggregation and further bilayer disruption by Aβ is [115].

Changes in lipid composition affect the fluidity, permeability, and lipid raft composition of the neuronal plasma membrane. The membranes from AD-diseased brain tissue differed from the healthy one in their nanoscale structure and were more susceptible to interaction with Aβ and its damaging effects [116]. The APP/PS1 transgenic mouse model of AD exhibited marked increase in lipid raft rigidity due to elevated levels of SM and decreased content of unsaturated fatty acids in the brain cortex [117]. Increased membrane order and viscosity of lipid rafts were observed in the frontal and entorhinal cortices of AD subjects [118]. Interestingly, mathematical modeling indicates that, during aging and AD progression, lipid rafts become larger, but the fluidity of non-raft regions of the plasma membrane increases. As Aβ toxicity is closely related to membrane microdomains, the enlargement of these structures may be connected with AD neuropathology [119,120]. Moreover, a comparative lipidomic study demonstrated elevations in SM and ganglioside GM3 levels in entorhinal cortex of AD patients [121].

## 3. Gangliosides and Aβ

### 3.1. Gangliosides

Glycosphingolipids are membrane molecules composed of a hydrophilic carbohydrate moiety and a hydrophobic ceramide part that contains a sphingosine and a fatty acid residue [103,122]. Glycosphingolipids play numerous physiological and pathophysiological roles in animal cells and tissues. They function as receptors for microbial toxins, mediators of cell adhesion, and modulators of signal transduction [123,124].

Gangliosides are sialic acid (N-acetylneuraminic)-containing glycosphingolipids localized primarily in the outer leaflet of the plasma membrane. Over 60 gangliosides have been characterized in tissues of vertebrates that differ in the position and number of sialic acid residues [125]. Gangliosides represent nearly 6% of the total lipid content in the brain [103], but, in the neuronal plasma membrane, their concentration can reach up to 15 mol [126]. Together with SM and cholesterol, gangliosides are the main components of membrane microdomains. Gangliosides reduce membrane fluidity due to lateral cooperative interactions between the ganglioside molecules. Even at a low concentration, gangliosides create clusters that are relevant to lipid rafts [30,127]. Blocking of ganglioside synthesis leads to destruction of lipid rafts and increased cell viability in cultured neurons exposed to Aβ oligomers, as well as less neurodegeneration in the cerebral cortex and improved spatial memory in AD model mice [128,129,130].

There are different forms of gangliosides participating in signal transduction, cell–cell recognition, and adhesion, especially in the developing nervous system [95,122,124]. Gangliosides GM1, GD1a, GD1b, and GT1b are the most common in the brain of mammals [131]. In brain tissue of patients with AD, the depletion of protective complex gangliosides GD1 and GM1 along with an increase in simple gangliosides GM2 and GM3 was observed [121,132,133]. Yet another study has revealed altered distribution of GM1 and GM2 gangliosides in AD compared with controls. These results support the idea of increased amounts of GM1 and GM2 in lipid rafts that would lead to the formation of toxic amyloid fibrils [31]. While total ganglioside amount in brains of AD humans decreases, specific populations concentrated in lipid rafts may increase [30,123,134,135]. Kim et al. [136] demonstrated that neither lipid rafts themselves, nor cholesterol as a key lipid of membrane microdomains, but gangliosides are necessary for Aβ aggregation.

### 3.2. GM1

The polar head of ganglioside GM1 contains four sugar residues and one molecule of sialic acid. Ganglioside GM1 is highly expressed throughout mammalian brain but is predominantly enriched within the white matter [131]. On the other hand, GM1 and GD2 are present on neuronal cell bodies contained within the grey matter [137]. In humans, the amount of complex gangliosides GM1 and GD1 increases 12–15 fold during development [122]. The GM1 clusters are enriched in the G1 stage in the neuronal cell membrane that explains the preferential Aβ binding to these membranes [82]. Aβ specifically binds to clusters of GM1, but not to membranes with uniformly distributed GM1. Clustering of GM1 is facilitated by cholesterol [13,92,138].

Increased levels of GM1 and GM2 gangliosides were found in lipid rafts isolated from the frontal and temporal cerebral cortex of AD individuals [30,31]. This elevation may facilitate the formation of toxic amyloid fibrils of Aβ, as increased GM1 levels were found in amyloid-positive neuritic terminals from AD cerebral cortex and apoE4 knock-in mouse brain during aging [139,140].

### 3.3. The Role of GM1 in Seeding and Accumulation of Aβ

Glycosphingolipids A complex of GM1 and Aβ (GAβ) has been identified in cerebral cortices from AD and Down′s syndrome subjects [91]. In the transgenic mouse model of AD, MALDI-IMS (imaging mass spectrometry) revealed brain-region specific accumulations of monosialogangliosides, including GM1, in the hippocampal and cortical amyloid plaques [141]. It was suggested that, after GM1 binding, the originally unordered amyloid peptide adopts an α-helical structure prior to its assembly into fibrils forming β-sheet structures [52,89,96,142,143,144]. Once the GAβ complex is formed, more soluble amyloid peptides join the aggregate and adopt a similar conformation. The original GAβ complex thus serves as a template for binding and conformation transition of Aβ [34,47,145]. It was found that especially ganglioside-enriched microdomains in the presynaptic neuronal membrane play a key role in the initiation of Aβ assembly [146]. Interactions between GM1 and Aβ involve hydrophobic interactions with membrane-embedded ceramide portion, electrostatic interactions, and hydrogen bonds with the hydrophilic sialic acid portion exposed on the outer membrane surface [142,147,148]. Ariga et al. demonstrated that, of several peptides tested, Aβ42 has the strongest affinity for GM1, whereas the less toxic Aβ40 binds to this ganglioside more weakly [59,149]. A recent in vivo model showed accelerated Aβ assembly in the brain of Drosophila expressing GM3 [150].

Aβ specifically recognizes ganglioside clusters through a glycosphingolipid-binding domain containing turn-inducing (Gly, Pro), basic (Arg, Lys, His), and aromatic residues (Phe, Tyr, Trp) [16]. NMR spectroscopy and MD simulations revealed two lysine residues (Lys16, Lys28) in Aβ responsible for GM1 binding. Simultaneously, binding of Aβ to lipid systems, not only gangliosides, is driven by the hydrophobic residues Val17–Ala21 [151]. For GM1 clusters recognition, the His13–Gln15 region is crucial, while binding of Lys18 to sialic acid triggers the helix formation at the C-terminus of Aβ. Other polar and hydrophobic interactions are necessary for finalizing the aggregation process of Aβ on a GM1-containing bilayer [148,152,153]. It was shown by Yamamoto et al. that aging and apo-E4 expression cooperatively accelerate Aβ aggregation in the brain through the increase and modulation of GM1 distribution in neuronal membranes [139]. The model of liposomes containing 10% *w*/*w* of cholesterol and 5% GM1 corroborated the importance of GM1 for Aβ–membrane interactions. After binding GM1, Aβ was able to produce perturbations in the lipid bilayer [154].

Among other gangliosides (GM1, GD1a, GD1b, and GT1b), GM1 seems to have the strongest seeding capacity [145]. As gangliosides are localized to lipid rafts, it was suggested that the GM1 clustering at presynaptic neuronal segments is a critical step for Aβ deposition in AD [144]. Matsubara et al. found that Aβ-sensitive ganglioside nanoclusters occur in synaptosomal membranes [155]. Computer simulations showed that Aβ perturbed palmitoyl-oleoyl-PC membrane structure, but inclusion of cholesterol and GM1 protected membrane from Aβ-induced disruption by increasing membrane rigidity. On the other hand, the carbohydrate headgroup of GM1 can act as an interaction partner for Aβ, leading to formation of toxic aggregates. Interestingly, binding of the amyloid peptide induced a β-hairpin structure at the C-terminus of the peptide that was not formed in the absence of the GM1 [152].

The dimerization of Aβ enhances the peptide hydrophobicity and enables Aβ42 oligomers to bind to GM1 ganglioside much more strongly than monomers, as was observed in membrane extracts of mouse hippocampus, as well as with in vitro binding assays [59]. On the other hand, fluorescence titration and biolayer interferometry experiments showed high-affinity binding of monomeric, but not oligomeric form of Aβ40 and Aβ42 to GM1-containing nanodiscs [156]. On the mouse model of AD, masking the sialic acid residue on GM1 with cholera toxin decreased Aβ oligomer-mediated LTP inhibition [59]. In transgenic mice that lacked all major brain gangliosides, significantly reduced amyloid deposition occurred and a decreased level of neurodegeneration and inflammation was determined. A similar effect was observed when the surface of gangliosides was blocked by a sialic acid-specific lectin [157].

**Figure 3:**
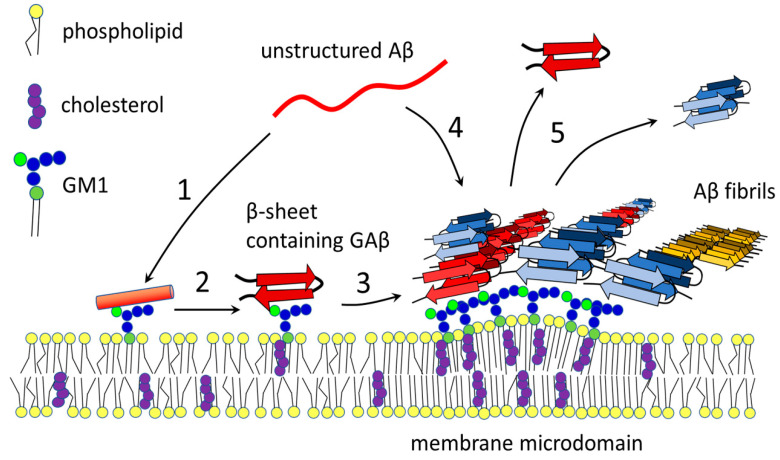
Aggregation of amyloid β on GM1-containing membrane. After processing of APP, Aβ (red) is released into intercellular space. Certain membrane molecules, including ganglioside GM1, serve as nucleation centers for Aβ aggregation. (1) Binding to non-clustered GM1 induces α-helical conformation in Aβ. (2) GM1 causes transition of α-helical to β-sheet structure. (3) Clusters of GM1 localized to membrane microdomains are responsible for concentration and aggregation of amyloid peptide into higher-molecular forms. Both parallel and antiparallel β-structures were observed in membrane bound amyloid fibrils. (4) Aggregates of Aβ serve as platforms for capturing and binding of monomers or oligomers circulating in the intercellular space. On the other hand, some portion of amyloid peptides may release from the aggregates (5). Adjusted according to [66,85,92,153,155].

Evangelisti et al. [10] found a quantitative relationship between the GM1 content in the cell membrane and the ability of the membrane to bind oligomers that cause toxic effects. Results from atomic force microscopy indicated that Aβ42 oligomers do not interact with membranes composed of PC and SM. On the other hand, GM1 is required for the peptide interaction with the membrane. This interaction, supported by cholesterol, leads to rapid membrane destruction [52,53]. Changes in local lipid composition during aging and progression of AD may induce the formation of ganglioside clusters that are recognized by Aβ. Then, Aβ undergoes a conformational transition to the β-sheet-rich structure that serves as a seed for toxic amyloid fibril formation [144] (Figure 3).

It was shown that GM1 can modulate binding of Aβ42 oligomers to artificial membranes, as well as Aβ-induced formation of membrane holes in a concentration-dependent manner [47]. Besides enhancing Aβ binding affinity, GM1 also causes a deeper penetration of this peptide into the lipid bilayer. The ability of Aβ to bind to the membrane is closely related to the clustering of GM1 and its specific location [158]. Aβ fibril formation on nanoclusters of GM1, but not of GM2, GD1, or GT1, was strongly induced in the presence of 10 mol % ganglioside, especially at cholesterol contents of 35–55 mol % [146]. In vitro studies on model membrane systems demonstrated that Aβ40 does not bind to isolated GM1 gangliosides but binds to GM1 clusters in membrane domains stabilized by cholesterol [36].

Multimodal imaging mass spectrometry showed that GM1–Aβ interactions links GM1 to mature amyloid aggregates associated with neurotoxic plaque formation [159]. Molecular dynamics simulations demonstrated the adhesion of Aβ40 to a GM1 cluster followed by helix formation, which is the initial stage of the pathological aggregation pathway [148]. Ikeda et al. [160] have demonstrated that the secondary structure and the mode of aggregation are dependent on the Aβ: GM1 ratio. They observed the transition of Aβ conformation from a random coil to an α-helix-rich structure after GM1 binding (at the Aβ: GM1 ratio of less than ∼0.013). With increasing levels of Aβ, peptide oligomers (15-mers) formed β-sheet structures and did not aggregate into fibrils. At Aβ/GM1 ratios above ∼0.044, the amyloid conformation was converted to a seed-prone β-structure that recruits monomers from the aqueous phase to form amyloid fibrils different from those formed in solution [160]. Density gradient ultracentrifugation used for separating the free from the bound peptide enabled Ahyayauch et al. to confirm that gangliosides facilitate the binding of Aβ42 to the bilayer and modify the peptide conformation to increase the β-sheet content [161].

Okada et al. [162] have recently found that Aβ fibrils bound to membranes are composed of mixed parallel and antiparallel β-sheets. The formation of the more toxic antiparallel β-sheet aggregates is supported by the Aβ–GM1 interaction. The peptide–lipid interaction results in the exposition of amyloid hydrophobic residues that make the fibrils sticky and adherent to membranes, thereby exacerbating the cytotocic effects of Aβ [162]. Importantly, the more hydrophobic environment facilitates the hydrogen bonding between Aβ40 molecules rather than between Aβ and other neighboring molecules, leading to the appearance of secondary structures and eventually amyloid fibrils [163]. On the other hand, using AFM Matsubara et al. found that Aβ typically self-assembles into antiparallel β-structures but by interacting with gangliosides the peptide can also form protofibrils with parallel β-sheets. These authors concluded that, by promoting the formation of parallel β-sheets, GM1 nanoclusters accelerate the elongation of Aβ fibrils [164]. Dai et al. [165] explored the role of GM1 present in vesicles with cholesterol and SM by the single molecule fluorescence tracking technique. GM1 induced the formation of Aβ42 fibrils even at low concentrations of the peptide. The amyloid peptides underwent a conformational transition from random coil structures to β-sheet-rich fibrils that were toxic to nerve cells [166]. MD simulations and NMR experiments indicated that two hydrophobic helical regions (residues 10–22 (β1) and residues 30–40 (β2)) of Aβ40 bound to the interface of GM1 micelles. Then, a β-sheet containing hairpin structure was formed by getting the β1 and β2 regions closer to each other. The β-hairpin structure can accelerate the formation of oligomers with the intermolecular β-sheet structure [166]. Interestingly, Fernández-Pérez et al. [82] observed that in GM1-rich microdomains of rat hippocampal neurons Aβ clustering led to perforation of the lipid bilayer.

Small unilamellar vesicles (SUV), composed of monosialogangliosides, cholesterol, and phospholipids were used as a model of lipid rafts in a study that mimicked intracellular environment by macromolecular crowding realized by the addition of polyvinylpyrrolidone (a high-molecular weight neutral polymer). The interaction between SUV and Aβ peptide proceeded mostly without affecting the membrane structure. On the other hand, in a crowding environment, the deformation of the SUV shape and Aβ peptide aggregation occurred [167].

### 3.4. The Effect of Other Sphingolipids and Cholesterol

There is some evidence that SM increases with age at presynaptic plasma membranes of mouse brain. SM is involved in the formation of unique membrane microdomains different from cholesterol-based lipid rafts, but enriched in gangliosides [144]. Under pathological conditions, the accumulation of both GM1 and SM in early endosomes leads to GM1 clustering responsible for GAβ formation, which results in GAβ-dependent amyloid fibril formation [35]. It was shown that intracellular Aβ42 aggregates form the nidus of eventual plaques, which are enlarged with the contribution of secreted Aβ [40]. It was suggested that GM1 cluster accumulation, causing GAβ generation, can occur following the internalization of GM1 into the endosomes [168].

The role of cholesterol in Aβ oligomerization seems to lie in the cholesterol ability to support the formation of GM1 clusters that preferentially interact with Aβ [36,103,145]. Cholesterol may help Aβ insert into membrane microdomains and gangliosides stabilize the toxic peptide species, such as protofibrils and oligomers, through hydrogen bonds, charged groups, and hydrophobic interactions [52,53,169]. Cholesterol molecules fill the space between GM1 molecules, forming hydrogen bonds with the ganglioside polar head. Specific conformation of ganglioside sugar moiety affected by cholesterol molecule plays a role in Aβ recognition and binding [170].

Cholesterol depletion significantly reduced Aβ accumulation on GM1 clusters in rat pheochromocytoma PC12 cells [88], while NGF-induced differentiation of PC12 cells increased both gangliosides and cholesterol and potentiated the accumulation and the cytotoxic effect of Aβ42 [11].

### 3.5. Clustering of GM1

The significance of GM1 clustering was stressed by Amaro et al. [92]. These authors used artificial lipid membranes and analyzed the effect of physiologically relevant concentrations of Aβ peptides and gangliosides. Single-molecule fluorescence techniques revealed triggering of Aβ40 oligomerization by SM and inhibition of Aβ40 aggregation by GM1 in the presence of nanomolar concentration of Aβ and 2–4 mol % of GM1. In contrast with the majority of other studies [96,97,118,120,145,171], they did not observe liquid-ordered phase characteristic for lipid rafts. In Amaro′s model, only fluid nanoscale GM1 clusters were monitored in the membrane, but these clusters did not induce Aβ oligomerization. Moreover, the presence of GM1 prevented the oligomerization of Aβ observed in dioleoyl-PC/cholesterol/SM membranes [92]. As this study was carried out at low ionic strength, electrostatic repulsion between negatively charged Aβ and anionic GM1 inhibiting the Aβ–GM1 interaction was much stronger than that at physiological ionic strength [153].

Cebecauer et al. proposed a model in which the local distribution of gangliosides, SM, and cholesterol play a role. This model indicates that non-raft nanoscopic GM1 organization might regulate ganglioside internalization via endocytosis. The local increase of GM1 concentration in endosomes may lead to enhanced GAβ formation that is connected to disruption of endosomal/lysosomal compartment [172]. However, similar effects could be connected to synaptosomes and rafts of neuronal cells, where the GM1 is enriched as well [140,144,173].

In dependence on the surface charge density on membranes, the Aβ peptides may attain different conformational states that have fundamental impact on the aggregation process of amyloidogenic proteins. Hence, the disease-related changes in ganglioside levels as well as its clustering may have a dramatic effect on Aβ-induced neurotoxicity and amyloid plaques formation [126].

While the aggregation effect of GM1 on Aβ is well known, it is important to mention the impact of Aβ binding on GM1 mobility in membrane. Single particle tracking experiments in living cells revealed that the membrane mobility of GM1 significantly decreased following the binding of Aβ42 aggregates to the plasma membrane. This finding indicates that amyloid aggregates may alter cellular processes dependent on the mobility and clustering of membrane rafts [174].

### 3.6. Neuroprotective Effect of GM1 in Neurodegeneration

Despite its potential to play a significant role in neurotoxic effects of Aβ, ganglioside GM1 is known to be strongly neuroprotective. Through interaction with membrane receptors, GM1 modifies cell differentiation, enhances responses to neurotrophic factors, and reduces cell damage induced by overstimulation of excitatory signaling pathways [135].

Neurotrophic and neuroprotective activities of GM1 have been well documented. Treatment with ganglioside stopped the progression of degenerative processes in AD patients [173,175]. GM1 also increased viability of PC12 cells exposed to Aβ that induced oxidative stress [176]. Yang et al. [177] reported that injection of GM1 into the hippocampus of AD rats can improve learning and memory deficits connected with Aβ-promoted oxidative damage. The neuroprotective function of GM1 was corroborated by a study where Na,K-ATPase activity was decreased in Aβ42-treated rats. GM1 was able to increase oxidant scavenging capacity of rat cerebral cortex and hippocampus tissue that led to a marked enhancement of Na,K-ATPase activity [178].

The effect of GM1 appears to be mediated by modulating some signal transduction systems, especially the tropomyosin-related kinase (Trk) receptors pathway [176]. Thus, GM1 can activate similar pathways as neurotrophins, including a modulatory role for ion channels and cellular Ca^2+^ homeostasis [125]. A different mechanism of antiamyloidogenic effect of GM1 lies in its ability to bind and sequester Aβ peptides, thereby preventing the formation of aggregates. GM1 in the blood binds Aβ what enables drawing Aβ out of the brain, because GAβ in the blood is not capable of crossing the blood–brain barrier and cannot be incorporated into plaques in the brain. Thus, peripheral administration of GM1 may be effective in reducing amyloid aggregation in AD by altering the Aβ blood/brain equilibrium [123,179]. In addition, administration of naked GM1 can decrease binding of amyloid fragments to neuronal lipid rafts [156]. Moreover, gangliosides may inhibit amyloidogenic processing of APP [180].

## 4. Conclusions

The Aβ peptide is known to be inherently unstable. Its spatial organization strongly depends on the surroundings. In solution, Aβ exists in an unordered conformation without any or with low participation of secondary structures, especially if it is in a monomer state. However, after oligomerization, Aβ adopts α-helical or β-sheet containing conformations, which reflects the formation of various hydrogen, electrostatic, dipole, and hydrophobic interactions between different parts of the peptide. When Aβ is incorporated into the hydrophobic lipid environment, different types of aggregates are formed than in water solution. Certain proteins and lipids can induce conformational changes in Aβ, which then aggregates and adopts different secondary, tertiary, and quaternary structures. As a result, oligomers of different organization and spatial arrangement are built in or on the plasma membrane depending on the local environment. Membrane microdomains corresponding to lipid rafts containing cholesterol, sphingomyelin, and ganglioside GM1 seem to represent main organizational centers for the formation of neurotoxic amyloid aggregates.

The high potential of Aβ to adopt different spatial arrangements is reflected by diverse impacts on target cells, where this peptide is attached. It is therefore very difficult to make general statements about the actions of Aβ. Transmembrane pores composed of α-helical or β-sheet secondary motives, as well as various surface-bound supramolecular structures displaying different spatial organization were observed in lipid bilayers. The intrinsic peptide instability makes the study of Aβ extremely hard because the experimental conditions inevitably influence Aβ conformation and aggregation. Nevertheless, marked progress in experimental techniques during past years led to a significant advance in understanding the pathophysiological processes induced by Aβ–membrane interactions. Among the most important partners of Aβ, ganglioside GM1 has been identified as the critical lipid molecule that drives the aggregation and deleterious effects of Aβ on neuronal cells. Importantly, cell membrane organization and lipid rafts can also play a role in these processes.

The plasma membrane directly affects the extent and mode of Aβ aggregation. As membrane phospholipids, cholesterol, and SM are widespread in the brain, they cannot be responsible for specific Aβ binding in the regions known to be the most damaged during AD. However, they can provide conditions for the facilitation of neurotoxic processes. It has been found that gangliosides are concentrated in membrane microdomains of synaptic regions. In particular, GM1 clusters serve as important platforms where Aβ is recognized, concentrated, and transformed into cell-damaging aggregates. The participation of cholesterol and other membrane-associated molecules may induce such spatial orientation of gangliosides which not only encourages the formation of membrane microdomains and promotes membrane stability, but also leads to the creating of specific interaction platforms for Aβ. Aβ bound to GM1 clusters may then adopt a specific conformation associated with neurotoxic effects observed in AD pathology.

For future research, it is of paramount importance to set up experimental conditions which would correspond as closely as possible to normal physiological milieu. In particular, the representation, concentration, and distribution of the key players, i.e., amyloid β and ganglioside GM1, their localization in lipid rafts or diffusion scattering in the plasma or endosomal membrane deserve the closest possible attention. In this context, brain organoids and neural stem cells derived from induced pluripotent stem cells may represent promising experimental models corresponding to the brain tissue of healthy subjects or AD patients. They are easily accessible to various experimental techniques and to manipulation of the levels of key substances, including GM1, cholesterol, and other membrane lipids.

## References

[1] Apostolova L.G. (2016). Alzheimer Disease. Continuum (Minneap. Minn.).

[2] GBD 2015 Disease and Injury Incidence and Prevalence Collaborators (2016). Global, regional, and national incidence, prevalence, and years lived with disability for 310 diseases and injuries, 1990–2015: A systematic analysis for the Global Burden of Disease Study 2015. Lancet.

[3] Chen X.Q., Mobley W.C. (2019). Exploring the Pathogenesis of Alzheimer Disease in Basal Forebrain Cholinergic Neurons: Converging Insights from Alternative Hypotheses. Front. Neurosci..

[4] Jan A., Adolfsson O., Allaman I., Buccarello A.L., Magistretti P.J., Pfeifer A., Muhs A., Lashuel H.A. (2011). Abeta42 neurotoxicity is mediated by ongoing nucleated polymerization process rather than by discrete Abeta42 species. J. Biol. Chem..

[5] Villemagne V.L., Burnham S., Bourgeat P., Brown B., Ellis K.A., Salvado O., Szoeke C., Macaulay S.L., Martins R., Maruff P. (2013). Amyloid beta deposition, neurodegeneration, and cognitive decline in sporadic Alzheimer’s disease: A prospective cohort study. Lancet Neurol..

[6] Hardy J., Selkoe D.J. (2002). The amyloid hypothesis of Alzheimer’s disease: Progress and problems on the road to therapeutics. Science.

[7] Selkoe D.J., Hardy J. (2016). The amyloid hypothesis of Alzheimer’s disease at 25 years. EMBO Mol. Med..

[8] Palmqvist S., Scholl M., Strandberg O., Mattsson N., Stomrud E., Zetterberg H., Blennow K., Landau S., Jagust W., Hansson O. (2017). Earliest accumulation of beta-amyloid occurs within the default-mode network and concurrently affects brain connectivity. Nat. Commun..

[9] Wong P.T., Schauerte J.A., Wisser K.C., Ding H., Lee E.L., Steel D.G., Gafni A. (2009). Amyloid-beta membrane binding and permeabilization are distinct processes influenced separately by membrane charge and fluidity. J. Mol. Biol..

[10] Evangelisti E., Cascella R., Becatti M., Marrazza G., Dobson C.M., Chiti F., Stefani M., Cecchi C. (2016). Binding affinity of amyloid oligomers to cellular membranes is a generic indicator of cellular dysfunction in protein misfolding diseases. Sci. Rep..

[11] Wakabayashi M., Matsuzaki K. (2007). Formation of amyloids by Abeta-(1-42) on NGF-differentiated PC12 cells: Roles of gangliosides and cholesterol. J. Mol. Biol..

[12] Amar F., Sherman M.A., Rush T., Larson M., Boyle G., Chang L., Gotz J., Buisson A., Lesne S.E. (2017). The amyloid-beta oligomer A beta*56 induces specific alterations in neuronal signaling that lead to tau phosphorylation and aggregation. Sci. Signal..

[13] Vahed M., Neya S., Matsuzaki K., Hoshino T. (2018). Analysis of Physicochemical Interaction of Abeta40 with a GM1 Ganglioside-Containing Lipid Membrane. J. Phys. Chem. B.

[14] Micelli S., Meleleo D., Picciarelli V., Gallucci E. (2004). Effect of sterols on beta-amyloid peptide (AbetaP 1-40) channel formation and their properties in planar lipid membranes. Biophys. J..

[15] Demuro A., Mina E., Kayed R., Milton S.C., Parker I., Glabe C.G. (2005). Calcium dysregulation and membrane disruption as a ubiquitous neurotoxic mechanism of soluble amyloid oligomers. J. Biol. Chem..

[16] Fantini J., Yahi N. (2010). Molecular insights into amyloid regulation by membrane cholesterol and sphingolipids: Common mechanisms in neurodegenerative diseases. Expert Rev. Mol. Med..

[17] Di Scala C., Chahinian H., Yahi N., Garmy N., Fantini J. (2014). Interaction of Alzheimer’s beta-amyloid peptides with cholesterol: Mechanistic insights into amyloid pore formation. Biochemistry.

[18] Sepulveda F.J., Fierro H., Fernandez E., Castillo C., Peoples R.W., Opazo C., Aguayo L.G. (2014). Nature of the neurotoxic membrane actions of amyloid-beta on hippocampal neurons in Alzheimer’s disease. Neurobiol. Aging.

[19] Revett T.J., Baker G.B., Jhamandas J., Kar S. (2013). Glutamate system, amyloid ss peptides and tau protein: Functional interrelationships and relevance to Alzheimer disease pathology. J. Psychiatry Neurosci..

[20] Kocahan S., Dogan Z. (2017). Mechanisms of Alzheimer’s Disease Pathogenesis and Prevention: The Brain, Neural Pathology, N-methyl-D-aspartate Receptors, Tau Protein and Other Risk Factors. Clin. Psychopharmacol. Neurosci..

[21] Baker-Nigh A., Vahedi S., Davis E.G., Weintraub S., Bigio E.H., Klein W.L., Geula C. (2015). Neuronal amyloid-beta accumulation within cholinergic basal forebrain in ageing and Alzheimer’s disease. Brain.

[22] Richter N., Beckers N., Onur O.A., Dietlein M., Tittgemeyer M., Kracht L., Neumaier B., Fink G.R., Kukolja J. (2018). Effect of cholinergic treatment depends on cholinergic integrity in early Alzheimer’s disease. Brain.

[23] Sultzer D.L. (2018). Cognitive ageing and Alzheimer’s disease: The cholinergic system redux. Brain.

[24] Machova E., Rudajev V., Smyckova H., Koivisto H., Tanila H., Dolezal V. (2010). Functional cholinergic damage develops with amyloid accumulation in young adult APPswe/PS1dE9 transgenic mice. Neurobiol. Dis..

[25] Janickova H., Rudajev V., Zimcik P., Jakubik J., Tanila H., El-Fakahany E.E., Dolezal V. (2013). Uncoupling of M1 muscarinic receptor/G-protein interaction by amyloid beta(1-42). Neuropharmacology.

[26] Janickova H., Rudajev V., Dolejsi E., Koivisto H., Jakubik J., Tanila H., El-Fakahany E.E., Dolezal V. (2015). Lipid-Based Diets Improve Muscarinic Neurotransmission in the Hippocampus of Transgenic APPswe/PS1dE9 Mice. Curr. Alzheimer Res..

[27] Ferreira-Vieira T.H., Guimaraes I.M., Silva F.R., Ribeiro F.M. (2016). Alzheimer’s disease: Targeting the Cholinergic System. Curr. Neuropharmacol..

[28] Viola K.L., Klein W.L. (2015). Amyloid beta oligomers in Alzheimer’s disease pathogenesis, treatment, and diagnosis. Acta Neuropathol..

[29] Chen G.F., Xu T.H., Yan Y., Zhou Y.R., Jiang Y., Melcher K., Xu H.E. (2017). Amyloid beta: Structure, biology and structure-based therapeutic development. Acta Pharmacol. Sin..

[30] Molander-Melin M., Blennow K., Bogdanovic N., Dellheden B., Mansson J.E., Fredman P. (2005). Structural membrane alterations in Alzheimer brains found to be associated with regional disease development; increased density of gangliosides GM1 and GM2 and loss of cholesterol in detergent-resistant membrane domains. J. Neurochem..

[31] Pernber Z., Blennow K., Bogdanovic N., Mansson J.E., Blomqvist M. (2012). Altered distribution of the gangliosides GM1 and GM2 in Alzheimer’s disease. Dement. Geriatr. Cogn. Disord..

[32] Arbor S.C., Lafontaine M., Cumbay M. (2016). Amyloid-beta Alzheimer targets—Protein processing, lipid rafts, and amyloid-beta pores. Yale J. Biol. Med..

[33] Owen M.C., Kulig W., Poojari C., Rog T., Strodel B. (2018). Physiologically-relevant levels of sphingomyelin, but not GM1, induces a beta-sheet-rich structure in the amyloid-beta(1-42) monomer. Biochim. Biophys. Acta Biomembr..

[34] Yanagisawa K. (2015). GM1 ganglioside and Alzheimer’s disease. Glycoconj. J..

[35] Yuyama K., Yanagisawa K. (2010). Sphingomyelin accumulation provides a favorable milieu for GM1 ganglioside-induced assembly of amyloid beta-protein. Neurosci. Lett..

[36] Kakio A., Nishimoto S., Yanagisawa K., Kozutsumi Y., Matsuzaki K. (2001). Cholesterol-dependent formation of GM1 ganglioside-bound amyloid beta-protein, an endogenous seed for Alzheimer amyloid. J. Biol. Chem..

[37] Kamenetz F., Tomita T., Hsieh H., Seabrook G., Borchelt D., Iwatsubo T., Sisodia S., Malinow R. (2003). APP processing and synaptic function. Neuron.

[38] Luhrs T., Ritter C., Adrian M., Riek-Loher D., Bohrmann B., Dobeli H., Schubert D., Riek R. (2005). 3D structure of Alzheimer’s amyloid-beta(1-42) fibrils. Proc. Natl. Acad. Sci. USA.

[39] Ahmed M., Davis J., Aucoin D., Sato T., Ahuja S., Aimoto S., Elliott J.I., Van Nostrand W.E., Smith S.O. (2010). Structural conversion of neurotoxic amyloid-beta(1-42) oligomers to fibrils. Nat. Struct. Mol. Biol..

[40] Gouras G.K. (2019). Aging, Metabolism, Synaptic Activity, and Abeta in Alzheimer’s Disease. Front. Aging Neurosci..

[41] Nasica-Labouze J., Nguyen P.H., Sterpone F., Berthoumieu O., Buchete N.V., Cote S., De Simone A., Doig A.J., Faller P., Garcia A. (2015). Amyloid beta Protein and Alzheimer’s Disease: When Computer Simulations Complement Experimental Studies. Chem. Rev..

[42] Verma M., Vats A., Taneja V. (2015). Toxic species in amyloid disorders: Oligomers or mature fibrils. Ann. Indian Acad. Neurol..

[43] Tran J., Chang D., Hsu F., Wang H., Guo Z. (2017). Cross-seeding between Abeta40 and Abeta42 in Alzheimer’s disease. FEBS Lett..

[44] Xue C., Tran J., Wang H., Park G., Hsu F., Guo Z. (2019). Abeta42 fibril formation from predominantly oligomeric samples suggests a link between oligomer heterogeneity and fibril polymorphism. R. Soc. Open Sci..

[45] Ono K., Tsuji M. (2020). Protofibrils of Amyloid-beta are Important Targets of a Disease-Modifying Approach for Alzheimer’s Disease. Int. J. Mol. Sci..

[46] Roychaudhuri R., Yang M., Hoshi M.M., Teplow D.B. (2009). Amyloid beta-protein assembly and Alzheimer disease. J. Biol. Chem..

[47] Williams T.L., Johnson B.R., Urbanc B., Jenkins A.T., Connell S.D., Serpell L.C. (2011). Abeta42 oligomers, but not fibrils, simultaneously bind to and cause damage to ganglioside-containing lipid membranes. Biochem. J..

[48] Bobo C., Chaignepain S., Henry S., Vignaud H., Ameadan A., Marchal C., Prado E., Doutch J., Schmitter J.M., Nardin C. (2017). Synthetic toxic Abeta1-42 oligomers can assemble in different morphologies. Biochim. Biophys. Acta Gen. Subj..

[49] Xue W.F., Hellewell A.L., Gosal W.S., Homans S.W., Hewitt E.W., Radford S.E. (2009). Fibril fragmentation enhances amyloid cytotoxicity. J. Biol. Chem..

[50] Lambert M.P., Barlow A.K., Chromy B.A., Edwards C., Freed R., Liosatos M., Morgan T.E., Rozovsky I., Trommer B., Viola K.L. (1998). Diffusible, nonfibrillar ligands derived from A beta(1-42) are potent central nervous system neurotoxins. Proc. Natl. Acad. Sci. USA.

[51] Lesne S., Koh M.T., Kotilinek L., Kayed R., Glabe C.G., Yang A., Gallagher M., Ashe K.H. (2006). A specific amyloid-beta protein assembly in the brain impairs memory. Nature.

[52] Yamamoto N., Matsubara E., Maeda S., Minagawa H., Takashima A., Maruyama W., Michikawa M., Yanagisawa K. (2007). A ganglioside-induced toxic soluble Abeta assembly. Its enhanced formation from Abeta bearing the Arctic mutation. J. Biol. Chem..

[53] Ewald M., Henry S., Lambert E., Feuillie C., Bobo C., Cullin C., Lecomte S., Molinari M. (2019). High speed atomic force microscopy to investigate the interactions between toxic Abeta1-42 peptides and model membranes in real time: Impact of the membrane composition. Nanoscale.

[54] Bernstein S.L., Dupuis N.F., Lazo N.D., Wyttenbach T., Condron M.M., Bitan G., Teplow D.B., Shea J.E., Ruotolo B.T., Robinson C.V. (2009). Amyloid-beta protein oligomerization and the importance of tetramers and dodecamers in the aetiology of Alzheimer’s disease. Nat. Chem..

[55] Lesne S.E., Sherman M.A., Grant M., Kuskowski M., Schneider J.A., Bennett D.A., Ashe K.H. (2013). Brain amyloid-beta oligomers in ageing and Alzheimer’s disease. Brain.

[56] Spencer R.K., Li H., Nowick J.S. (2014). X-ray crystallographic structures of trimers and higher-order oligomeric assemblies of a peptide derived from Abeta(17-36). J. Am. Chem. Soc..

[57] Cline E.N., Bicca M.A., Viola K.L., Klein W.L. (2018). The Amyloid-beta Oligomer Hypothesis: Beginning of the Third Decade. J. Alzheimers Dis..

[58] Esparza T.J., Zhao H., Cirrito J.R., Cairns N.J., Bateman R.J., Holtzman D.M., Brody D.L. (2013). Amyloid-beta oligomerization in Alzheimer dementia versus high-pathology controls. Ann. Neurol..

[59] Hong S., Ostaszewski B.L., Yang T., O’malley T.T., Jin M., Yanagisawa K., Li S., Bartels T., Selkoe D.J. (2014). Soluble Abeta oligomers are rapidly sequestered from brain ISF in vivo and bind GM1 ganglioside on cellular membranes. Neuron.

[60] Jeong J.S., Ansaloni A., Mezzenga R., Lashuel H.A., Dietler G. (2013). Novel mechanistic insight into the molecular basis of amyloid polymorphism and secondary nucleation during amyloid formation. J. Mol. Biol..

[61] Tycko R. (2015). Amyloid polymorphism: Structural basis and neurobiological relevance. Neuron.

[62] Qiang W., Yau W.M., Lu J.X., Collinge J., Tycko R. (2017). Structural variation in amyloid-beta fibrils from Alzheimer’s disease clinical subtypes. Nature.

[63] Petkova A.T., Leapman R.D., Guo Z., Yau W.M., Mattson M.P., Tycko R. (2005). Self-propagating, molecular-level polymorphism in Alzheimer’s beta-amyloid fibrils. Science.

[64] Niu Z., Zhang Z., Zhao W., Yang J. (2018). Interactions between amyloid beta peptide and lipid membranes. Biochim. Biophys. Acta Biomembr..

[65] Rasmussen J., Mahler J., Beschorner N., Kaeser S.A., Hasler L.M., Baumann F., Nystrom S., Portelius E., Blennow K., Lashley T. (2017). Amyloid polymorphisms constitute distinct clouds of conformational variants in different etiological subtypes of Alzheimer’s disease. Proc. Natl. Acad. Sci. USA.

[66] Larini L., Shea J.E. (2012). Role of beta-Hairpin Formation in Aggregation: The Self-Assembly of the Amyloid-beta(25-35) Peptide. Biophys. J..

[67] Nag S., Sarkar B., Chandrakesan M., Abhyanakar R., Bhowmik D., Kombrabail M., Dandekar S., Lerner E., Haas E., Maiti S. (2013). A folding transition underlies the emergence of membrane affinity in amyloid-beta. Phys. Chem. Chem. Phys..

[68] Wei G., Shea J.E. (2006). Effects of solvent on the structure of the Alzheimer amyloid-beta(25-35) peptide. Biophys. J..

[69] Choo L.P., Wetzel D.L., Halliday W.C., Jackson M., Levine S.M., Mantsch H.H. (1996). In situ characterization of beta-amyloid in Alzheimer’s diseased tissue by synchrotron Fourier transform infrared microspectroscopy. Biophys. J..

[70] Shao H., Jao S., Ma K., Zagorski M.G. (1999). Solution structures of micelle-bound amyloid beta-(1-40) and beta-(1-42) peptides of Alzheimer’s disease. J. Mol. Biol..

[71] Vivekanandan S., Brender J.R., Lee S.Y., Ramamoorthy A. (2011). A partially folded structure of amyloid-beta(1-40) in an aqueous environment. Biochem. Biophys. Res. Commun..

[72] Bhowmik D., Maclaughlin C.M., Chandrakesan M., Ramesh P., Venkatramani R., Walker G.C., Maiti S. (2014). pH changes the aggregation propensity of amyloid-beta without altering the monomer conformation. Phys. Chem. Chem. Phys..

[73] Lockhart C., Klimov D.K. (2014). Alzheimer’s Abeta10-40 peptide binds and penetrates DMPC bilayer: An isobaric-isothermal replica exchange molecular dynamics study. J. Phys. Chem. B.

[74] Tycko R. (2016). Molecular Structure of Aggregated Amyloid-beta: Insights from Solid-State Nuclear Magnetic Resonance. Cold Spring Harb. Perspect. Med..

[75] Jang H., Arce F.T., Ramachandran S., Capone R., Lal R., Nussinov R. (2010). beta-Barrel topology of Alzheimer’s beta-amyloid ion channels. J. Mol. Biol..

[76] Schmidt M., Sachse C., Richter W., Xu C., Fandrich M., Grigorieff N. (2009). Comparison of Alzheimer Abeta(1-40) and Abeta(1-42) amyloid fibrils reveals similar protofilament structures. Proc. Natl. Acad. Sci. USA.

[77] Xiao Y., Ma B., Mcelheny D., Parthasarathy S., Long F., Hoshi M., Nussinov R., Ishii Y. (2015). Abeta(1-42) fibril structure illuminates self-recognition and replication of amyloid in Alzheimer’s disease. Nat. Struct. Mol. Biol..

[78] Barz B., Olubiyi O.O., Strodel B. (2014). Early amyloid beta-protein aggregation precedes conformational change. Chem. Commun. (Camb.).

[79] Vignaud H., Bobo C., Lascu I., Sorgjerd K.M., Zako T., Maeda M., Salin B., Lecomte S., Cullin C. (2013). A structure-toxicity study of Ass42 reveals a new anti-parallel aggregation pathway. PLoS ONE.

[80] Bonhommeau S., Talaga D., Hunel J., Cullin C., Lecomte S. (2017). Tip-Enhanced Raman Spectroscopy to Distinguish Toxic Oligomers from Abeta1-42 Fibrils at the Nanometer Scale. Angew. Chem. Int. Ed. Engl..

[81] Davis C.H., Berkowitz M.L. (2010). A molecular dynamics study of the early stages of amyloid-beta(1-42) oligomerization: The role of lipid membranes. Proteins.

[82] Fernandez-Perez E.J., Sepulveda F.J., Peoples R., Aguayo L.G. (2017). Role of membrane GM1 on early neuronal membrane actions of Abeta during onset of Alzheimer’s disease. Biochim. Biophys. Acta Mol. Basis Dis..

[83] Simakova O., Arispe N.J. (2007). The cell-selective neurotoxicity of the Alzheimer’s Abeta peptide is determined by surface phosphatidylserine and cytosolic ATP levels. Membrane binding is required for Abeta toxicity. J. Neurosci..

[84] Mclaurin J., Chakrabartty A. (1996). Membrane disruption by Alzheimer beta-amyloid peptides mediated through specific binding to either phospholipids or gangliosides. Implications for neurotoxicity. J. Biol. Chem..

[85] Korshavn K.J., Bhunia A., Lim M.H., Ramamoorthy A. (2016). Amyloid-beta adopts a conserved, partially folded structure upon binding to zwitterionic lipid bilayers prior to amyloid formation. Chem. Commun. (Camb.).

[86] Yates E.A., Owens S.L., Lynch M.F., Cucco E.M., Umbaugh C.S., Legleiter J. (2013). Specific domains of Abeta facilitate aggregation on and association with lipid bilayers. J. Mol. Biol..

[87] Sasahara K., Morigaki K., Shinya K. (2013). Effects of membrane interaction and aggregation of amyloid beta-peptide on lipid mobility and membrane domain structure. Phys. Chem. Chem. Phys..

[88] Ji S.R., Wu Y., Sui S.F. (2002). Cholesterol is an important factor affecting the membrane insertion of beta-amyloid peptide (A beta 1-40), which may potentially inhibit the fibril formation. J. Biol. Chem..

[89] Wakabayashi M., Okada T., Kozutsumi Y., Matsuzaki K. (2005). GM1 ganglioside-mediated accumulation of amyloid beta-protein on cell membranes. Biochem. Biophys. Res. Commun..

[90] Nicholson A.M., Ferreira A. (2009). Increased membrane cholesterol might render mature hippocampal neurons more susceptible to beta-amyloid-induced calpain activation and tau toxicity. J. Neurosci..

[91] Yanagisawa K., Odaka A., Suzuki N., Ihara Y. (1995). GM1 ganglioside-bound amyloid beta-protein (A beta): A possible form of preamyloid in Alzheimer’s disease. Nat. Med..

[92] Amaro M., Sachl R., Aydogan G., Mikhalyov I., Vacha R., Hof M. (2016). GM1 Ganglioside Inhibits beta-Amyloid Oligomerization Induced by Sphingomyelin. Angew. Chem. Int. Ed. Engl..

[93] Zhou X., Xu J. (2012). Free cholesterol induces higher beta-sheet content in Abeta peptide oligomers by aromatic interaction with Phe19. PLoS ONE.

[94] Van Weering J.R.T., Scheper W. (2019). Endolysosome and Autolysosome Dysfunction in Alzheimer’s Disease: Where Intracellular and Extracellular Meet. CNS Drugs.

[95] Yu X., Zheng J. (2012). Cholesterol promotes the interaction of Alzheimer beta-amyloid monomer with lipid bilayer. J. Mol. Biol..

[96] Matsuzaki K., Kato K., Yanagisawa K. (2010). Abeta polymerization through interaction with membrane gangliosides. Biochim. Biophys. Acta.

[97] Mori K., Mahmood M.I., Neya S., Matsuzaki K., Hoshino T. (2012). Formation of GM1 ganglioside clusters on the lipid membrane containing sphingomyeline and cholesterol. J. Phys. Chem. B.

[98] Ahmed S.N., Brown D.A., London E. (1997). On the origin of sphingolipid/cholesterol-rich detergent-insoluble cell membranes: Physiological concentrations of cholesterol and sphingolipid induce formation of a detergent-insoluble, liquid-ordered lipid phase in model membranes. Biochemistry.

[99] Anderson R.G., Jacobson K. (2002). A role for lipid shells in targeting proteins to caveolae, rafts, and other lipid domains. Science.

[100] Pike L.J. (2004). Lipid rafts: Heterogeneity on the high seas. Biochem. J..

[101] Garner A.E., Smith D.A., Hooper N.M. (2008). Visualization of detergent solubilization of membranes: Implications for the isolation of rafts. Biophys. J..

[102] Sonnino S., Aureli M., Mauri L., Ciampa M.G., Prinetti A. (2015). Membrane lipid domains in the nervous system. Front. Biosci. (Landmark Ed.).

[103] Haughey N.J., Bandaru V.V., Bae M., Mattson M.P. (2010). Roles for dysfunctional sphingolipid metabolism in Alzheimer’s disease neuropathogenesis. Biochim. Biophys. Acta.

[104] Lingwood D., Simons K. (2010). Lipid Rafts as a Membrane-Organizing Principle. Science.

[105] Lisanti M.P., Scherer P.E., Tang Z., Sargiacomo M. (1994). Caveolae, Caveolin and Caveolin-Rich Membrane Domains: A Signalling Hypothesis. Trends Cell Biol..

[106] Eckert G.P., Igbavboa U., Muller W.E., Wood W.G. (2003). Lipid rafts of purified mouse brain synaptosomes prepared with or without detergent reveal different lipid and protein domains. Brain Res..

[107] Moravcova Z., Rudajev V., Stohr J., Novotny J., Cerny J., Parenti M., Milligan G., Svoboda P. (2004). Long-term agonist stimulation of IP prostanoid receptor depletes the cognate G(s)alpha protein in membrane domains but does not change the receptor level. Biochim. Biophys. Acta.

[108] Matousek P., Novotny J., Rudajev V., Svoboda P. (2005). Prolonged agonist stimulation does not alter the protein composition of membrane domains in spite of dramatic changes induced in a specific signaling cascade. Cell Biochem. Biophys..

[109] Rudajev V., Novotny J., Hejnova L., Milligan G., Svoboda P. (2005). Dominant portion of thyrotropin-releasing hormone receptor is excluded from lipid domains. Detergent-resistant and detergent-sensitive pools of TRH receptor and Gqalpha/G11alpha protein. J. Biochem..

[110] Chakrabarti S., Chang A., Gintzler A.R. (2010). Subcellular localization of mu-opioid receptor G(s) signaling. J. Pharmacol. Exp. Ther..

[111] Lauren J., Gimbel D.A., Nygaard H.B., Gilbert J.W., Strittmatter S.M. (2009). Cellular prion protein mediates impairment of synaptic plasticity by amyloid-beta oligomers. Nature.

[112] Rushworth J.V., Hooper N.M. (2010). Lipid Rafts: Linking Alzheimer’s Amyloid-beta Production, Aggregation, and Toxicity at Neuronal Membranes. Int. J. Alzheimers Dis..

[113] Staneva G., Puff N., Stanimirov S., Tochev T., Angelova M.I., Seigneuret M. (2018). The Alzheimer’s disease amyloid-beta peptide affects the size-dynamics of raft-mimicking Lo domains in GM1-containing lipid bilayers. Soft Matter.

[114] Azouz M., Cullin C., Lecomte S., Lafleur M. (2019). Membrane domain modulation of Abeta1-42 oligomer interactions with supported lipid bilayers: An atomic force microscopy investigation. Nanoscale.

[115] Terakawa M.S., Lin Y., Kinoshita M., Kanemura S., Itoh D., Sugiki T., Okumura M., Ramamoorthy A., Lee Y.H. (2018). Impact of membrane curvature on amyloid aggregation. Biochim. Biophys. Acta Biomembr..

[116] Drolle E., Negoda A., Hammond K., Pavlov E., Leonenko Z. (2017). Changes in lipid membranes may trigger amyloid toxicity in Alzheimer’s disease. PLoS ONE.

[117] Fabelo N., Martin V., Marin R., Santpere G., Aso E., Ferrer I., Diaz M. (2012). Evidence for premature lipid raft aging in APP/PS1 double-transgenic mice, a model of familial Alzheimer disease. J. Neuropathol. Exp. Neurol..

[118] Fabelo N., Martin V., Marin R., Moreno D., Ferrer I., Diaz M. (2014). Altered lipid composition in cortical lipid rafts occurs at early stages of sporadic Alzheimer’s disease and facilitates APP/BACE1 interactions. Neurobiol. Aging.

[119] Malchiodi-Albedi F., Contrusciere V., Raggi C., Fecchi K., Rainaldi G., Paradisi S., Matteucci A., Santini M.T., Sargiacomo M., Frank C. (2010). Lipid raft disruption protects mature neurons against amyloid oligomer toxicity. Biochim. Biophys. Acta.

[120] Santos G., Diaz M., Torres N.V. (2016). Lipid Raft Size and Lipid Mobility in Non-raft Domains Increase during Aging and Are Exacerbated in APP/PS1 Mice Model of Alzheimer’s Disease. Predictions from an Agent-Based Mathematical Model. Front. Physiol..

[121] Chan R.B., Oliveira T.G., Cortes E.P., Honig L.S., Duff K.E., Small S.A., Wenk M.R., Shui G., Di Paolo G. (2012). Comparative lipidomic analysis of mouse and human brain with Alzheimer disease. J. Biol. Chem..

[122] Yu R.K., Nakatani Y., Yanagisawa M. (2009). The role of glycosphingolipid metabolism in the developing brain. J. Lipid Res..

[123] Ariga T., Mcdonald M.P., Yu R.K. (2008). Role of ganglioside metabolism in the pathogenesis of Alzheimer’s disease—A review. J. Lipid Res..

[124] Yagi-Utsumi M., Kato K. (2015). Structural and dynamic views of GM1 ganglioside. Glycoconj. J..

[125] Magistretti P.J., Geisler F.H., Schneider J.S., Li P.A., Fiumelli H., Sipione S. (2019). Gangliosides: Treatment Avenues in Neurodegenerative Disease. Front. Neurol..

[126] Mojumdar E.H., Grey C., Sparr E. (2019). Self-Assembly in Ganglioside-Phospholipid Systems: The Co-Existence of Vesicles, Micelles, and Discs. Int. J. Mol. Sci..

[127] Sonnino S., Mauri L., Chigorno V., Prinetti A. (2007). Gangliosides as components of lipid membrane domains. Glycobiology.

[128] Ohmi Y., Tajima O., Ohkawa Y., Yamauchi Y., Sugiura Y., Furukawa K., Furukawa K. (2011). Gangliosides are essential in the protection of inflammation and neurodegeneration via maintenance of lipid rafts: Elucidation by a series of ganglioside-deficient mutant mice. J. Neurochem..

[129] Herzer S., Meldner S., Rehder K., Grone H.J., Nordstrom V. (2016). Lipid microdomain modification sustains neuronal viability in models of Alzheimer’s disease. Acta Neuropathol. Commun..

[130] Herzer S., Hagan C., Von Gerichten J., Dieterle V., Munteanu B., Sandhoff R., Hopf C., Nordstrom V. (2018). Deletion of Specific Sphingolipids in Distinct Neurons Improves Spatial Memory in a Mouse Model of Alzheimer’s Disease. Front. Mol. Neurosci..

[131] Vajn K., Viljetic B., Degmecic I.V., Schnaar R.L., Heffer M. (2013). Differential distribution of major brain gangliosides in the adult mouse central nervous system. PLoS ONE.

[132] Fukami Y., Ariga T., Yamada M., Yuki N. (2017). Brain Gangliosides in Alzheimer’s Disease: Increased Expression of Cholinergic Neuron-Specific Gangliosides. Curr. Alzheimer Res..

[133] Caughlin S., Maheshwari S., Agca Y., Agca C., Harris A.J., Jurcic K., Yeung K.K., Cechetto D.F., Whitehead S.N. (2018). Membrane-lipid homeostasis in a prodromal rat model of Alzheimer’s disease: Characteristic profiles in ganglioside distributions during aging detected using MALDI imaging mass spectrometry. Biochim. Biophys. Acta Gen. Subj..

[134] Hicks D.A., Nalivaeva N.N., Turner A.J. (2012). Lipid rafts and Alzheimer’s disease: Protein-lipid interactions and perturbation of signaling. Front. Physiol..

[135] Chiricozzi E., Lunghi G., Di Biase E., Fazzari M., Sonnino S., Mauri L. (2020). GM1 Ganglioside Is A Key Factor in Maintaining the Mammalian Neuronal Functions Avoiding Neurodegeneration. Int. J. Mol. Sci..

[136] Kim S.I., Yi J.S., Ko Y.G. (2006). Amyloid beta oligomerization is induced by brain lipid rafts. J. Cell. Biochem..

[137] Marconi S., De Toni L., Lovato L., Tedeschi E., Gaetti L., Acler M., Bonetti B. (2005). Expression of gangliosides on glial and neuronal cells in normal and pathological adult human brain. J. Neuroimmunol..

[138] Matsuzaki K. (2014). How do membranes initiate Alzheimer’s Disease? Formation of toxic amyloid fibrils by the amyloid beta-protein on ganglioside clusters. Acc. Chem. Res..

[139] Yamamoto N., Igbabvoa U., Shimada Y., Ohno-Iwashita Y., Kobayashi M., Wood W.G., Fujita S.C., Yanagisawa K. (2004). Accelerated Abeta aggregation in the presence of GM1-ganglioside-accumulated synaptosomes of aged apoE4-knock-in mouse brain. FEBS Lett..

[140] Gylys K.H., Fein J.A., Yang F., Miller C.A., Cole G.M. (2007). Increased cholesterol in Abeta-positive nerve terminals from Alzheimer’s disease cortex. Neurobiol. Aging.

[141] Kaya I., Jennische E., Dunevall J., Lange S., Ewing A.G., Malmberg P., Baykal A.T., Fletcher J.S. (2020). Spatial Lipidomics Reveals Region and Long Chain Base Specific Accumulations of Monosialogangliosides in Amyloid Plaques in Familial Alzheimer’s Disease Mice (5xFAD) Brain. ACS Chem. Neurosci..

[142] Choo-Smith L.P., Surewicz W.K. (1997). The interaction between Alzheimer amyloid beta(1-40) peptide and ganglioside GM1-containing membranes. FEBS Lett..

[143] Hayashi H., Kimura N., Yamaguchi H., Hasegawa K., Yokoseki T., Shibata M., Yamamoto N., Michikawa M., Yoshikawa Y., Terao K. (2004). A seed for Alzheimer amyloid in the brain. J. Neurosci..

[144] Yamamoto N., Matsubara T., Sato T., Yanagisawa K. (2008). Age-dependent high-density clustering of GM1 ganglioside at presynaptic neuritic terminals promotes amyloid beta-protein fibrillogenesis. Biochim. Biophys. Acta.

[145] Kakio A., Nishimoto S., Yanagisawa K., Kozutsumi Y., Matsuzaki K. (2002). Interactions of amyloid beta-protein with various gangliosides in raft-like membranes: Importance of GM1 ganglioside-bound form as an endogenous seed for Alzheimer amyloid. Biochemistry.

[146] Matsubara T., Nishihara M., Yasumori H., Nakai M., Yanagisawa K., Sato T. (2017). Size and Shape of Amyloid Fibrils Induced by Ganglioside Nanoclusters: Role of Sialyl Oligosaccharide in Fibril Formation. Langmuir.

[147] Chi E.Y., Frey S.L., Lee K.Y. (2007). Ganglioside G(M1)-mediated amyloid-beta fibrillogenesis and membrane disruption. Biochemistry.

[148] Tachi Y., Okamoto Y., Okumura H. (2019). Conformational Change of Amyloid-beta 40 in Association with Binding to GM1-Glycan Cluster. Sci. Rep..

[149] Ariga T., Kobayashi K., Hasegawa A., Kiso M., Ishida H., Miyatake T. (2001). Characterization of high-affinity binding between gangliosides and amyloid beta-protein. Arch. Biochem. Biophys..

[150] Yamasaki Y., Tsuda L., Suzuki A., Yanagisawa K. (2018). Induction of ganglioside synthesis in Drosophila brain accelerates assembly of amyloid beta protein. Sci. Rep..

[151] Bera S., Korshavn K.J., Kar R.K., Lim M.H., Ramamoorthy A., Bhunia A. (2016). Biophysical insights into the membrane interaction of the core amyloid-forming Abeta40 fragment K16-K28 and its role in the pathogenesis of Alzheimer’s disease. Phys. Chem. Chem. Phys..

[152] Manna M., Mukhopadhyay C. (2013). Binding, conformational transition and dimerization of amyloid-beta peptide on GM1-containing ternary membrane: Insights from molecular dynamics simulation. PLoS ONE.

[153] Matsuzaki K. (2020). Abeta-ganglioside interactions in the pathogenesis of Alzheimer’s disease. Biochim. Biophys. Acta Biomembr..

[154] Nicastro M.C., Spigolon D., Librizzi F., Moran O., Ortore M.G., Bulone D., Biagio P.L., Carrotta R. (2016). Amyloid beta-peptide insertion in liposomes containing GM1-cholesterol domains. Biophys. Chem..

[155] Matsubara T., Iijima K., Yamamoto N., Yanagisawa K., Sato T. (2013). Density of GM1 in nanoclusters is a critical factor in the formation of a spherical assembly of amyloid beta-protein on synaptic plasma membranes. Langmuir.

[156] Thomaier M., Gremer L., Dammers C., Fabig J., Neudecker P., Willbold D. (2016). High-Affinity Binding of Monomeric but Not Oligomeric Amyloid-beta to Ganglioside GM1 Containing Nanodiscs. Biochemistry.

[157] Dukhinova M., Veremeyko T., Yung A.W.Y., Kuznetsova I.S., Lau T.Y.B., Kopeikina E., Chan A.M.L., Ponomarev E.D. (2019). Fresh evidence for major brain gangliosides as a target for the treatment of Alzheimer’s disease. Neurobiol. Aging.

[158] Mikhalyov I., Olofsson A., Grobner G., Johansson L.B. (2010). Designed fluorescent probes reveal interactions between amyloid-beta(1-40) peptides and GM1 gangliosides in micelles and lipid vesicles. Biophys. J..

[159] Michno W., Wehrli P.M., Zetterberg H., Blennow K., Hanrieder J. (2019). GM1 locates to mature amyloid structures implicating a prominent role for glycolipid-protein interactions in Alzheimer pathology. Biochim. Biophys. Acta Proteins Proteom..

[160] Ikeda K., Yamaguchi T., Fukunaga S., Hoshino M., Matsuzaki K. (2011). Mechanism of amyloid beta-protein aggregation mediated by GM1 ganglioside clusters. Biochemistry.

[161] Ahyayauch H., De La Arada I., Masserini M.E., Arrondo J.L.R., Goni F.M., Alonso A. (2020). The Binding of Abeta42 Peptide Monomers to Sphingomyelin/Cholesterol/Ganglioside Bilayers Assayed by Density Gradient Ultracentrifugation. Int. J. Mol. Sci..

[162] Okada Y., Okubo K., Ikeda K., Yano Y., Hoshino M., Hayashi Y., Kiso Y., Itoh-Watanabe H., Naito A., Matsuzaki K. (2019). Toxic Amyloid Tape: A Novel Mixed Antiparallel/Parallel beta-Sheet Structure Formed by Amyloid beta-Protein on GM1 Clusters. ACS Chem. Neurosci..

[163] Fukunaga S., Ueno H., Yamaguchi T., Yano Y., Hoshino M., Matsuzaki K. (2012). GM1 cluster mediates formation of toxic Abeta fibrils by providing hydrophobic environments. Biochemistry.

[164] Matsubara T., Yasumori H., Ito K., Shimoaka T., Hasegawa T., Sato T. (2018). Amyloid-beta fibrils assembled on ganglioside-enriched membranes contain both parallel beta-sheets and turns. J. Biol. Chem..

[165] Dai Y., Zhang M., Shi X., Wang K., Gao G., Shen L., Sun T. (2020). Kinetic study of Abeta(1-42) amyloidosis in the presence of ganglioside-containing vesicles. Colloids Surf. B Biointerfaces.

[166] Itoh S.G., Yagi-Utsumi M., Kato K., Okumura H. (2019). Effects of a Hydrophilic/Hydrophobic Interface on Amyloid-beta Peptides Studied by Molecular Dynamics Simulations and NMR Experiments. J. Phys. Chem. B.

[167] Hirai M., Ajito S., Sato S., Ohta N., Igarashi N., Shimizu N. (2018). Preferential Intercalation of Human Amyloid-beta Peptide into Interbilayer Region of Lipid-Raft Membrane in Macromolecular Crowding Environment. J. Phys. Chem. B.

[168] Yuyama K., Yanagisawa K. (2009). Late endocytic dysfunction as a putative cause of amyloid fibril formation in Alzheimer’s disease. J. Neurochem..

[169] Fantini J., Yahi N., Garmy N. (2013). Cholesterol accelerates the binding of Alzheimer’s beta-amyloid peptide to ganglioside GM1 through a universal hydrogen-bond-dependent sterol tuning of glycolipid conformation. Front. Physiol..

[170] Mao Y., Shang Z., Imai Y., Hoshino T., Tero R., Tanaka M., Yamamoto N., Yanagisawa K., Urisu T. (2010). Surface-induced phase separation of a sphingomyelin/cholesterol/ganglioside GM1-planar bilayer on mica surfaces and microdomain molecular conformation that accelerates Abeta oligomerization. Biochim. Biophys. Acta.

[171] Yanagisawa K. (2011). Pathological significance of ganglioside clusters in Alzheimer’s disease. J. Neurochem..

[172] Cebecauer M., Hof M., Amaro M. (2017). Impact of GM1 on Membrane-Mediated Aggregation/Oligomerization of beta-Amyloid: Unifying View. Biophys. J..

[173] Svennerholm L. (1994). Gangliosides--a new therapeutic agent against stroke and Alzheimer’s disease. Life Sci..

[174] Calamai M., Pavone F.S. (2013). Partitioning and confinement of GM1 ganglioside induced by amyloid aggregates. FEBS Lett..

[175] Svennerholm L., Brane G., Karlsson I., Lekman A., Ramstrom I., Wikkelso C. (2002). Alzheimer disease—Effect of continuous intracerebroventricular treatment with GM1 ganglioside and a systematic activation programme. Dement. Geriatr. Cogn. Disord..

[176] Sokolova T.V., Zakharova I.O., Furaev V.V., Rychkova M.P., Avrova N.F. (2007). Neuroprotective effect of ganglioside GM1 on the cytotoxic action of hydrogen peroxide and amyloid beta-peptide in PC12 cells. Neurochem. Res..

[177] Yang R., Wang Q., Min L., Sui R., Li J., Liu X. (2013). Monosialoanglioside improves memory deficits and relieves oxidative stress in the hippocampus of rat model of Alzheimer’s disease. Neurol. Sci..

[178] Kreutz F., Scherer E.B., Ferreira A.G.K., Petry F.D., Pereira C.L., Santana F., Wyse A.T.D., Salbego C.G., Trindade V.M.T. (2013). Alterations on Na^+^,K^+^-ATPase and Acetylcholinesterase Activities Induced by Amyloid-beta Peptide in Rat Brain and GM1 Ganglioside Neuroprotective Action. Neurochem. Res..

[179] Matsuoka Y., Saito M., Lafrancois J., Saito M., Gaynor K., Olm V., Wang L., Casey E., Lu Y., Shiratori C. (2003). Novel therapeutic approach for the treatment of Alzheimer’s disease by peripheral administration of agents with an affinity to beta-amyloid. J. Neurosci..

[180] Tsai Y.F., Yang D.J., Ngo T.H., Shih C.H., Wu Y.F., Lee C.K., Phraekanjanavichid V., Yen S.F., Kao S.H., Lee H.M. (2019). Ganglioside Hp-s1 Analogue Inhibits Amyloidogenic Toxicity in Alzheimer’s Disease Model Cells. ACS Chem. Neurosci..

